# Pre-impact fall detection using an inertial sensor unit

**DOI:** 10.1186/1757-1146-7-S1-A124

**Published:** 2014-04-08

**Authors:** Soonjae Ahn, Isu Shin, Youngho Kim

**Affiliations:** 1 Department of Biomedical Engineering and Institute of Medical Engineering, Yonsei University, Wonju, Gangwon, 220-710, Korea

## 

Falls are a major cause of injuries and deaths in older adults [1]. As for intervention strategies, one of the important problems in preventing or reducing the severity of injury in the elderly is to detect falls in its descending phase before the impact [2]. If a fall can be detected in its earliest stage in the descending phase, more efficient impact reduction systems can be implemented with a longer lead-time for minimizing injury [3,4]. In this study, we implemented a pre-impact fall detection algorithm using an inertial sensor unit.

Totally, forty male volunteers participated in the experiment (three types of falls and seven types of ADLs). An inertia sensor unit, placed at waist, was used to measure subject’s acceleration, angular velocity and vertical angle during various activities. In order to detect pre-impact, the threshold of acceleration and angular velocity was set to 0.8g and 30°/s, respectively, based on the data from the first twenty subjects. Furthermore, the threshold of vertical angle was set to 30° because the maximum angle in the ADL did not exceed 30°. This fall detection algorithm was evaluated for another twenty subjects.

The results showed that both acceleration and angular velocity during three different falls were greater than the threshold during several ADLs and the vertical angle did not exceed 30°. The vertical angle exceeded 30° only during sit-lying activity, but the acceleration did not reach 0.8g (Table 1). Based on the pre-impact fall detection algorithm, no false detection was found (100% sensitivity) for all falls.

**Table 1:** Peak acceleration, angular velocity and vertical angle during falls and ADLs.

Trials		Acceleration (g)	Angular velocity (°/s)		Angle (°)	
			
			Pitch	Roll	Sagittal	Lateral
**Falls**	**Backward**	4.1 ± 0.6	300.3 ± 59.7	45.7 ± 14.2	94.2 ± 4.7	4.7 ± 3.2
	**Forward**	4.5 ± 0.5	220.6 ± 41.6	75.9 ± 17.2	89.6 ± 9.2	8.4 ± 4.6
	**Side**	4.4 ± 0.6	121.2 ± 13.7	419.4 ± 61.3	6.69 ± 2.7	75.95 ± 12.4

**ADLs**	**Sit-Stand**	1.4 ± 0.2	110 ± 23.1	8.9 ± 7.6	31.1 ± 6.1	2.6 ± 1.3
	**Stand-Sit**	2.2 ± 0.3	392.3 ± 61.3	11.2 ± 6.1	11.63 ± 3.1	1.12 ± 2.7
	**Sit-Lying**	1.1 ± 0.1	80.7 ± 31.7	15.3 ± 3.8	90.3 ± 8.3	4.3 ± 2.8
	**Walking**	2.1 ± 0.2	50.1 ± 10.9	59.3 ± 14.9	1.4 ± 6.2	2.1 ± 3.1
	**Jump**	7.5 ± 1.1	421.2 ± 149.1	102.3 ± 62.1	27.3 ± 2.1	3.6 ± 3.8
	**Running**	4.2 ± 0.9	132.8 ± 45.7	98.2 ± 34.9	11.5 ± 9.7	2.6 ± 4.1

Furthermore, no incorrect detection was found (100% specificity) for all ADLs. The lead time was 474 ± 38.3ms, 590.3 ± 122.6ms and 527 ± 62.3ms in the backward, the forward and the side falls, respectively.

In this study, a pre-impact fall detection algorithm was developed using an inertial sensor unit. The present pre-impact fall detection algorithm can be implemented with a wearable fall injury minimization system to track a user’s body movement.
